# Minimizing stroke risk in off-pump CABG: the role of clampless devices and the piggyback proximal anastomosis technique

**DOI:** 10.3389/fcvm.2025.1555394

**Published:** 2025-03-03

**Authors:** Massimo Baudo, Francesco Cabrucci, Amanda Yakobitis, Courtney Murray, Gianluca Torregrossa

**Affiliations:** ^1^ Department of Cardiac Surgery Research, Lankenau Institute for Medical Research, Main Line Health, Wynnewood, PA, United States; ^2^ Department of Cardiac Surgery, Lankenau Heart Institute, Lankenau Medical Center, Main Line Health, Wynnewood, PA, United States

**Keywords:** proximal anastomosis, CABG, stroke, OPCABG, piggyback, aortic manipulation

## Abstract

**Introduction:**

Numerous techniques have been developed to minimize risk of perioperative stroke during coronary artery bypass grafting (CABG), including off-pump approach, preoperative and intraoperative imaging of the ascending aorta (CT scan and epiaortic ultrasound), anaortic CABG with bilateral internal thoracic artery, clampless devices for the construction of proximal anastomosis and minimal aortic manipulation with a single aortic inflow for all proximal grafts (piggyback proximal anastomosis). The aim of this study was to evaluate the clinical outcomes of CABG patients who underwent off pump CABG with proximal anastomosis constructed with the use of a clampless device and in a piggyback fashion.

**Methods:**

This observational study included 112 consecutive patients undergoing CABG with the piggyback proximal technique at the Lankenau Heart Institute between June 2021 and January 2024. Primary endpoints included overall mortality, cardiac-related mortality, stroke, myocardial infarction, repeat revascularization. Intraoperative transit time flow measurement (TTFM) was also analyzed.

**Results:**

The mean age of the cohort was 67.8 ± 8.7 years, with 75.9% (85/112) being male. All patients underwent off-pump CABG. The piggyback anastomosis consisted of vein-on-vein (52.7%, 59/112), artery-on-vein (43.8%, 49/112), and double vein/artery configurations (3.6%, 4/112). Postoperatively, no strokes occurred. At 30 days no patient died or required repeat revascularization. The mean hospital stay was 5.5 [4.0–8.0] days. At a mean follow-up of 1.0 [0.5–1.7] years, no cardiac deaths were recorded, with an overall survival of 98.2% (110/112). Repeat piggyback revascularization was 3.6% (4/112) at a mean of 2.0 ± 0.5 years. TTFM demonstrated superior flow rates in artery-on-vein grafts [50 (40–70) ml/min] compared to vein-on-vein grafts [40 (30–53.5), *p* < 0.001].

**Conclusions:**

When a proximal anastomosis cannot be avoided during off pump CABG, the combination of a piggyback proximal anastomosis together with the use of a clampless aortic device, demonstrated promising early mid-term outcomes almost nullifying the perioperative risk of clinical stroke. Intraoperative TTFM showed excellent flow rates, especially when arterial grafts were used. The technique is a viable option in high-risk patients with severe aortic disease, offering a safe and effective approach to multivessel revascularization with minimal aortic manipulation. Further studies with longer follow-up are warranted to confirm its long-term benefits.

## 1 Introduction

Coronary artery bypass grafting (CABG) remains the most effective revascularization strategy for patients with advanced coronary artery disease (CAD). Despite its success in reducing mortality and improving cardiac function, CABG carries inherent risks, most notably stroke and postoperative cognitive decline (1, 2). Cerebrovascular accidents, which include overt strokes and silent brain infarcts (SBI) that lack discernible neurological symptoms, can significantly impact postoperative outcomes. These SBI may accumulate over time, potentially contributing to long-term cognitive decline in CABG patients (3, 4).

Since SYNTAX trial that reported a stroke rate of 2.2% (5), minimizing aortic manipulation has become a key strategy in reducing the incidence of stroke during CABG (6). The aorta is a primary source of atheromatous debris, which can embolize during surgery and lead to cerebrovascular events. To mitigate this risk, several techniques have been developed, including single aortic cross clamp to complete proximal anastomosis during on pump CABG, off pump anaortic CABG using bilateral thoracic arteries for inflow (7), preoperative CT scans to assess aortic calcification, and intraoperative epiaortic ultrasound to identify the optimal site for aortic puncture (8, 9). In addition to these methods, two more techniques should be incorporated into the coronary surgeon's armamentarium.

First, the piggyback proximal anastomosis technique, where multiple grafts are attached through a single anastomotic site on the ascending aorta (10, 11). This approach reduces the number of aortic punctures and manipulation, lowering the risk of atheroembolism and potentially decreasing the incidence of both clinical and SBI. In addition to neuroprotection, the piggyback technique provides a stable anastomotic site that supports the use of a second arterial conduit, such as the radial artery or right internal thoracic artery (RITA) in free-graft configuration, preventing kinking or flattening that may occur with direct aortic anastomosis (10, 11).

Second, the use of clampless proximal anastomosis devices, avoiding the need for aortic side-biting clamp, offers a valid solutions for safer and more efficient aortic anastomoses during off-pump CABG (OPCABG) or during on pump beating heart CABG. These devices aim to reduce the risk of cerebral embolic complications associated with aortic clamping, particularly in patients with a high burden of atherosclerotic disease (12).

This study aimed to evaluate early mid-term clinical outcomes in patients undergoing OPCABG with clampless devices and the piggyback proximal aortic-coronary graft anastomosis technique, providing data to demonstrate that using a clampless single hole for all proximal anastomoses is safe, reproducible, and beneficial. Specifically, this approach may reduce stroke risk, enhance graft flow, and improve graft patency, particularly when a second arterial conduit is used. We hypothesize that this method may also contribute to better cognitive outcomes postoperatively.

## 2 Materials and methods

The study protocol received approval from the Main Line Health Hospitals Institutional Review Board (IRB 45CFR164.512). Given the retrospective nature of the study, individual patient consent was waived. This was an observational study in which all consecutive patients undergoing OPCABG with the piggyback proximal technique at Lankenau Heart Institute (Lankenau Medical Center, Wynnewood, PA, USA) between June 2021 and January 2024 were enrolled. All piggyback anastomoses were performed by a single surgeon. Patients were included by all demographics and preoperative characteristics.

The primary objective was to evaluate the incidence of stroke, overall mortality, cardiac-related mortality, cardiac readmissions, myocardial infarction, and the need for repeat target revascularization in patients undergoing OPCABG utilizing clampless devices and the piggyback proximal anastomosis technique.

Secondary endpoints included the transit time flow measurements (TTFM) of the aortocoronary grafts in different piggyback configurations: vein-on-vein, artery-on-vein, double vein and artery.

### 2.1 Surgical technique and “aortic safety culture”

All patients underwent a preoperative non-contrast CT scan of the chest to evaluate the burden, extent, and location of aortic wall calcification. Additionally, irrespective of the CT findings, intraoperative epiaortic ultrasound was systematically performed to assess the aortic wall quality.

**Figure 1:**
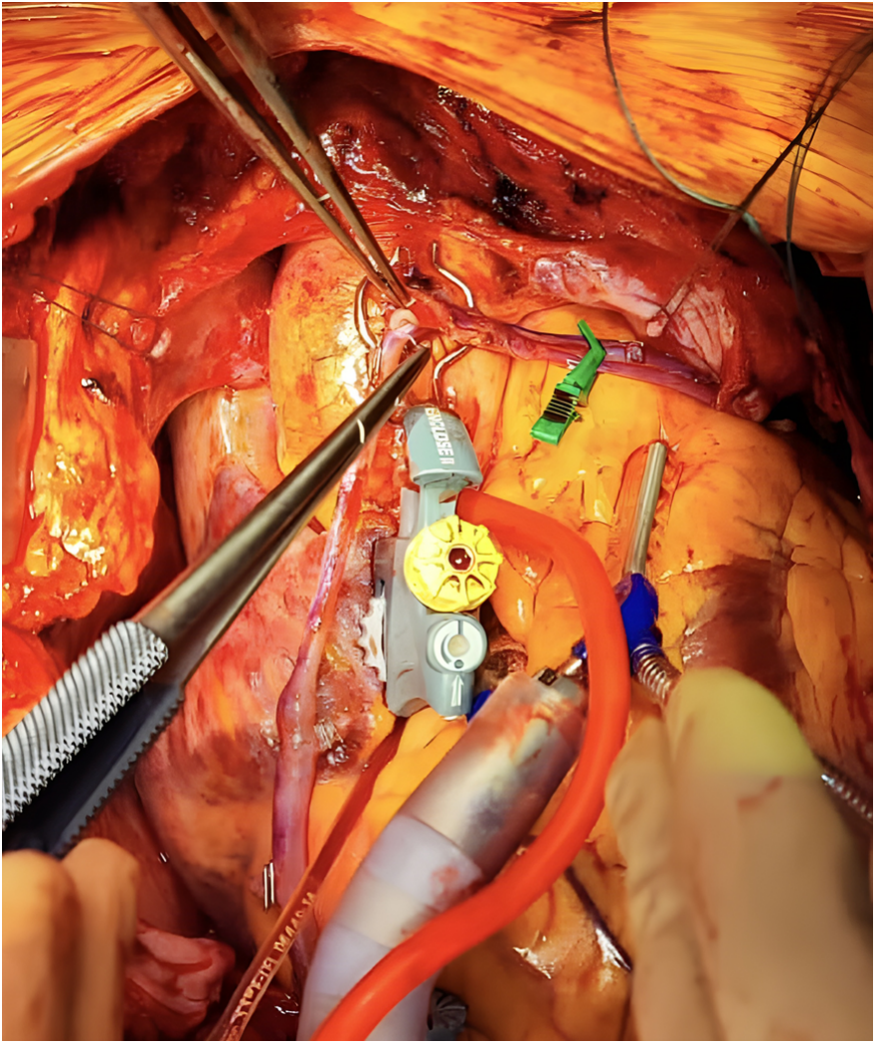
Intraoperative creation of a piggyback anastomosis with a proximal anastomosis device.

When extensive aortic calcification is detected the preferred approach is anaortic OPCABG to minimize aortic manipulation. In cases where a proximal anastomosis is deemed feasible, a clampless device is consistently utilized to mitigate the atheroembolic risk. Specifically, when the aortic wall is thickened with scattered calcifications involving the posterior wall, the Heartstring III Proximal Seal System® (Getinge, Sweden) device is favored for its adaptability. Conversely, when the aortic wall is more accessible and less burdened by calcification, the Enclose® II (Peters Surgical, USA) device is preferred, as it provides superior sealing (Figure 1). A comprehensive description of the technique for using clampless devices is beyond the scope of this paper and has been previously detailed (13).

After creating a single hole in the ascending aorta with one of the aforementioned devices, a segment of the saphenous vein graft (SVG) is first anastomosed to the aorta in the usual fashion using a 6.0 polypropylene suture. Following our institutional culture of safety, the first proximal anastomosis—the foundation of the piggyback configuration—is consistently secured using a CorKnot®Micro (LSI Solution) (14). This approach facilitates subsequent identification and selective catheterization of the aortocoronary grafts by interventional cardiologists, thereby ensuring optimal visualization when required.

**Figure 2:**
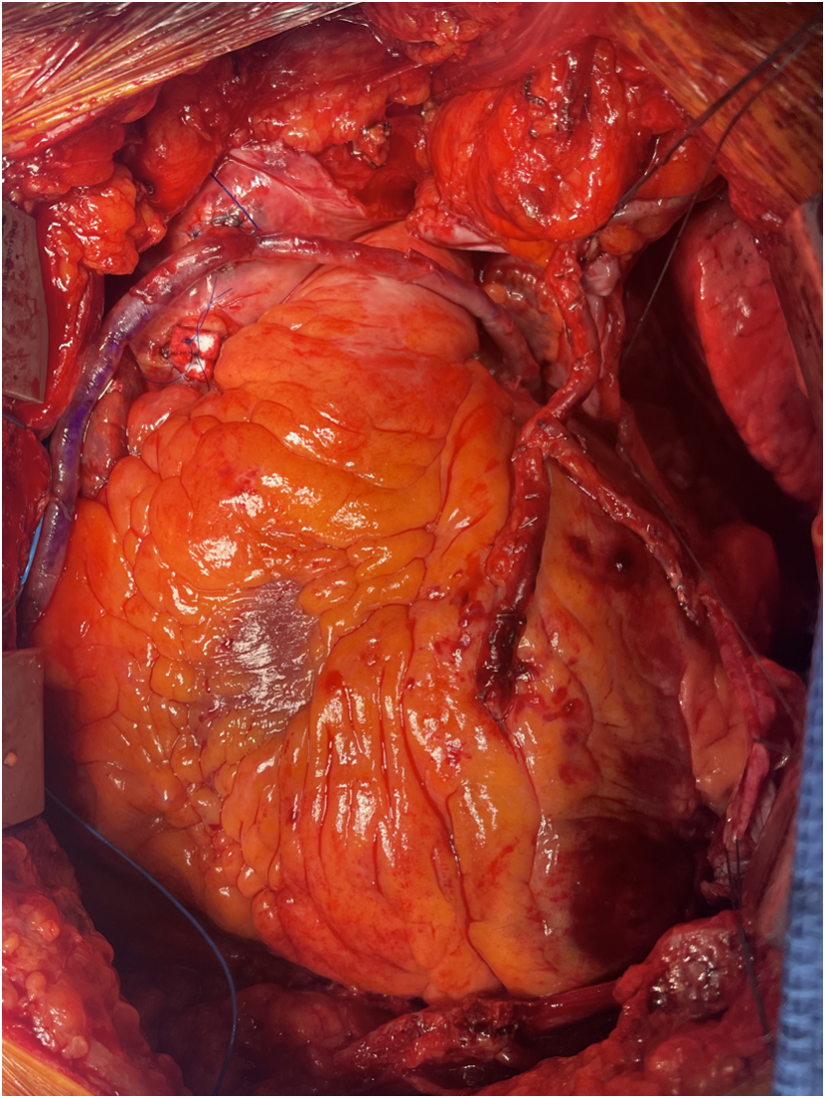
Piggyback anastomosis example.

Instead of punching a second hole in the aorta, the hood of the SVG, directly over the aortotomy, is longitudinally incised with a scalpel to create an orifice. A second graft, either another SVG or an arterial conduit, is then anastomosed to the hood of the first anastomosis using a 7.0 or 8.0 polypropylene suture, completing the piggyback configuration (Figure 2).

Preventing postoperative strokes is paramount, given their potential to negate the benefits of surgical revascularization and their long-term sequelae, including subtle sensory, motor, and memory impairments. A clampless piggyback technique is advocated as part of a broader strategy to minimize aortic manipulation and reduce stroke risk. Recognizing that routine postoperative neuroimaging for asymptomatic patients is not always feasible, a dedicated protocol has been established to identify patients at high risk for strokes. This protocol integrates preoperative factors (e.g., aortic wall calcification burden, atrial fibrillation, carotid atherosclerosis, history of cerebrovascular events, reduced ejection fraction) with intra- and postoperative variables [e.g., prolonged hypotension during OPCABG or in the intensive care unit (ICU), low hemoglobin levels] to determine a threshold of clinical suspicion. In the ICU, thorough neurological evaluations upon sedation cessation, followed by continuous monitoring, and systematic assessments in the step-down unit enable prompt neuroimaging when changes in sensory, motor, or neurocognitive function are detected. This comprehensive approach provides a framework for the early detection of both overt and SBI.

### 2.2 Statistical analysis

Categorical variables were expressed as numbers and percentages. The Kolmogorov–Smirnov test was used to assess normal distribution. Continuous variables are presented as mean and standard deviation (SD) if normally distributed and compared using the student's t-test or ANOVA accordingly. While they were presented as median and interquartile range (IQR) if not normally distributed and compared using the Mann–Whitney U test or Kruskal–Wallis test. Microsoft Office Excel program (Microsoft, Redmond, Washington) was used for data extraction and all analyses were performed in R, version 4.3.1 (R Software for Statistical Computing) within RStudio.

## 3 Results

A total of 112 patients were enrolled and patient's baseline characteristics were summarized in Table 1. The mean age was 67.8 ± 8.7 and 85 (75.9%) patients were men. More than 90% of patients suffered from hypertension (104/112), 44 (39.3%) from diabetes, and 51 (45.5%) from dyslipidemia. Previous percutaneous coronary interventions were performed in 29 (25.9%) patients, while 2 (1.8%) had a previous cardiac surgery. The mean ejection fraction was 54.9% ± 11.8%, and the median STS-PROM was 1.04% [IQR: 0.64–1.92].

**Table 1:** Preoperative patient's characteristics.

Patient characteristics	*N* = 112
Male, *n* (%)	85 (75.9)
Age, years, mean (SD)	67.8 (8.7)
BMI, kg/m^2^, (mean (SD)	28.8 (5.3)
STS PROM, %, median [IQR]	1.04 [0.64–1.92]
Hypertension, *n* (%)	104 (92.9)
Dyslipidemia, *n* (%)	51 (45.5)
Diabetes, *n* (%)	44 (39.3)
Hb1Ac, %, mean (SD)	6.6 (1.5)
Creatinine, mg/dl, mean (SD)	1.0 [0.8–1.1]
Dialysis, *n* (%)	3 (2.7)
History of smoke, *n* (%)	66 (58.9)
Chronic lung disease, *n* (%)	26 (23.2)
Prior cerebrovascular accident, *n* (%)	10 (8.9)
Peripheral vascular disease, *n* (%)	20 (17.9)
Prior myocardial infarction, *n* (%)	54 (48.2)
Redo chest, *n* (%)	2 (1.8)
Prior PCI, *n* (%)	29 (25.9)
Atrial fibrillation, *n* (%)	15 (13.4)
Prior pacemaker, *n* (%)	2 (1.8)
Diseased coronaries, *n* (%)	
Two	13 (11.6)
Three	95 (84.8)
Four	4 (3.6)
Ejection fraction, %, mean (SD)	54.9 (11.8)

BMI, body mass index; IQR, interquartile range; PCI, percutaneous coronary intervention; SD, standard deviation; STS PROM, Society of Thoracic Surgeons predicted risk of mortality.

All patients underwent OPCABG, and the mean number of anastomoses was 3.4 ± 0.6. The radial artery was the most common (54/112, 48.2%) second arterial graft. A Y-graft, I-graft, and sequential grafting were used 15 (13.4%), 8 (7.1%), and 17 (15.2%) times respectively, Table 2. Overall, in 33 patients (29.5%) a concomitant procedure was performed. The full list of procedures is listed in Table 2.

**Table 2:** Intraoperative outcomes.

Intraoperative outcomes	Patients *N* = 112
OPCABG, *n* (%)	112 (100)
Total number of anastomoses, mean ± SD	3.4 ± 0.6
Number of anastomoses	
2	5 (4.5)
3	64 (57.1)
4	40 (35.7)
5	2 (1.8)
6	1 (0.9)
LITA to LAD, *n* (%)	108 (96.4)
RITA to LAD, *n* (%)	2 (1.8)
Second arterial, *n* (%)	65 (58.0)
RAD *n* (%)	54 (48.2)
RITA *n* (%)	10 (8.9)
Total arterial, *n* (%)	5 (4.5)
SVG, *n* (%)	111 (99.1)
Y graft, *n* (%)	15 (13.4)
I graft, *n* (%)	8 (7.1)
Sequential, *n* (%)	17 (15.2)
Concomitant procedures	
AtriClip, *n* (%)	33 (29.5)
PM/ICD implant, *n* (%)	1 (0.9)
Piggyback composition	
Vein on vein, *n* (%)	59 (52.7)
Artery on vein, *n* (%)	49 (43.8)
Double vein and artery, *n* (%)	4 (3.6)

AF, atrial fibrillation; AV, aortic valve; CABG, coronary artery bypass grafting; LITA , left internal thoracic artery; OPCABG, off-pump CABG; PM/ICD, pacemaker/implantable cardioverter defibrillator; RITA, right internal thoracic artery.

As far as the piggyback composition is concerned, 59 (52.7%) patients received a vein-on-vein piggyback anastomosis, 49 (43.8%) an artery-on-vein, and 4 (3.6%) a double vein and artery.

### 3.1 Primary endpoints

**Figure 3:**
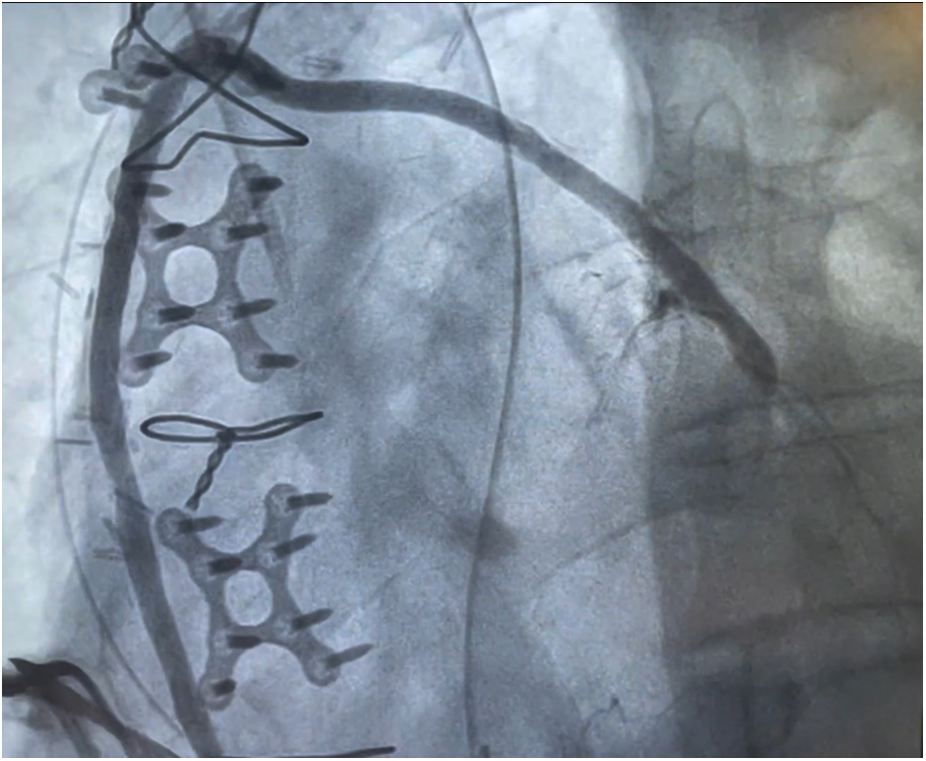
Postoperative angiographic visualization of the piggyback proximal anastomosis.

Two (1.8%) patients required ventilation longer than 24 h. Importantly, no strokes or myocardial infarctions occurred, and only one (0.9%) patient needed surgical re-exploration for bleeding. The mean length of hospital stay was 5.5 [4.0–8.0] days. At 30 days, no patient died or required repeat revascularization. Cardiac readmission occurred in 3 (2.7%) patients, Table 3. Patients who underwent postoperative angiography demonstrated no evidence of thrombosis, occlusion, kinking, or torsion of the piggyback anastomoses (Figure 3). Furthermore, the feasibility of angiographic assessment of the piggyback technique was confirmed, as interventional cardiologists experienced no difficulties in identifying or injecting the piggyback proximal anastomosis during the procedure. At a mean follow-up of 1.0 [0.5–1.7] years, there were no cardiac-related deaths, with an overall survival of 98.2% (110/112). Only one myocardial infarction occurred (0.9%), and four strokes (3.6%) were reported. In this regard, one stroke occurred in a patient with a known arteriovenous malformation that eventually ruptured, and one confirmed after a subsequent percutaneous coronary intervention, thus not directly related to the CABG surgery or anastomotic technique. Repeat Piggyback revascularization rate was 3.6% (4/112) at a mean of 2.0 ± 0.5 years. Among the revascularized grafts, three were veins and one was an artery. As far as the piggyback configuration of these patients who underwent percutaneous interventions, revascularization was performed on two grafts attached directly to the aorta and two grafts placed on top of the first graft, thus showing that repeat revascularization is independent of the piggyback configuration, Table 4.

**Table 3:** Postoperative outcomes.

Postoperative outcomes	*N* = 112
Extubated in OR, *n* (%)	42 (37.5)
Ventilation time, hours, median [IQR]	3.8 [3.0–6.0]
Ventilation >24 h, *n* (%)	2 (1.8)
Stroke, *n* (%)	0 (0.0)
Bleeding requiring surgery, *n* (%)	1 (0.9)
Myocardial infarction, *n* (%)	0 (0.0)
Repeat target revascularization <24 h, *n* (%)	0 (0.0)
Dialysis, *n* (%)	1 (0.9)
Atrial fibrillation, *n* (%)	27 (24.1)
Length of stay, days, median [IQR]	5.5 [4.0–8.0]
30-day mortality, *n* (%)	0 (0.0)
30-day repeat target revascularization, *n* (%)	0 (0.0)
30-day cardiac readmission, *n* (%)	3 (2.7)
Follow-up outcomes	*N* = 112
Follow-up time, years, median [IQR]	1.0 [0.5–1.7]
Overall survival, *n* (%)	110 (98.2)
Cardiac death, *n* (%)	0 (0.0)
Repeat piggyback revascularization, *n* (%)	4 (3.6)
Acute myocardial infarction, *n* (%)	1 (0.9)
Stroke, *n* (%)	4 (3.6)

IQR, interquartile range; OR, operating room; RBC, red blood cells.

**Table 4:** Repeat revascularization analysis.

Patient	CABG strategy	Repeat revascularization vessel	Years from surgery
1	OPCABG X 4 LITA LAD, SVG (Ao) OM1, SVG Diag (off SVG OM1), SVG PDA	OM1 – Piggyback	2.04
2	OPCABG X 4 LITA LAD, SVG (Ao) Diag, SVG (off SVG Diag) RI, SVG (Ao) PDA	RI – Piggyback	2.28
3	OPCABG X 3 LITA LAD, SVG (Ao) PDA, LRAD (off SVG) OM	RCA – Piggyback	1.30
4	OPCABG X 3 LITA LAD, LRAD (off SVG) PDA, SVG (Ao) OM	RCA – Piggyback	1.44

Ao, aorta; LAD, left anterior descending; Diag, diagonal; LITA, left internal thoracic artery; LRAD, left radial artery; OM, obtuse marginal; OPCABG, off-pump coronary artery bypass grafting; PDA, posterior descending artery; RCA, right coronary artery; RI, ramus intermedius; SVG, saphenous vein graft.

### 3.2 Secondary endpoint: transit time flow measurement

Coronary flow at transit time flow measurement of the left internal thoracic (LITA) – left anterior descending (LAD) was 50 [38–75] ml/min. The TTFM of the piggyback proximal anastomosis is described in Table 5. All grafts showed sufficient flow to their target coronary. The second graft demonstrated superior flow rates, especially when an artery was utilized, as compared to either a second SVG graft or the first SVG graft (*p* = 0.003). This was particularly true considering the posterior descending artery (PDA), *p* = 0.019. The median pulsatility index for the vein-on-vein, artery-on-vein, and vein-on-aorta were 2.1 [2.0–2.8], 2.0 [1.8–2.0], and 2.5 [2.0–3.0], *p* = 0.026, respectively. It is conceivable that the arterial conduit (from an artery-on-vein piggyback configuration) exhibited a higher average flow compared to the venous conduit (from a vein-on-vein piggyback configuration) due to a selection bias in conduit choice based on target vessel characteristics. The surgical strategy prioritizes the use of the radial artery for chronic total occlusions (CTOs) or vessels with a very high degree of stenosis—particularly the posterior descending artery (PDA)—while a venous conduit is typically employed for vessels with a lower degree of stenosis. Consequently, the higher runoff in the target vessel receiving the arterial conduit may account for the increased TTFM values observed.

**Table 5:** Intraoperative TTFM flows on the piggyback proximal anastomosis.

Piggyback	Overall	OM	Diagonal	PDA	*p*-value
Vein on vein (ml/min) median (IQR)	40 [30–53.5]	40 [30–52]	35 [21.5–52.5]	30 [28–65]	Total: 0.893 Pairwise OM vs. DIAG: 0.390 OM vs. PDA: 0.603 DIAG vs. PDA: 0.826
Artery on vein (ml/min) median (IQR)	50 [40–70]	45 [40–60]	–	67.5 [53.8–98.8]	***p*** **=** **0.013**
Vein on aorta (ml/min) median (IQR)	45 [30–65]	35 [28–58]	57.5 [40–72.5]	50 [30–67.5]	Total: 0.081 Pairwise OM vs. DIAG: 0.110 OM vs. PDA: **0.044** DIAG vs. PDA: 0.516
*p*-value	Total: **0.003** Pairwise VoV vs. AoV: **<** **0.001** VoV vs. VoAo: 0.131 AoV vs. VoAo: **0.025**	Total: **0.019** Pairwise VoV vs. AoV: **0.043** VoV vs. VoAo: 0.459 AoV vs. VoAo: **0.009**	*p* = 0.063	Total: **0.019** Pairwise VoV vs. AoV: **0.024** VoV vs. VoAo: 0.254 AoV vs. VoAo: **0.013**	

DIAG, diagonal coronary artery; IQR, interquartile range; OM, obtuse marginal; PDA, posterior descending artery; TTFM, transit time flow measurement.

Bold values denote statistical significance at the *p* < 0.05 level.

The artery-on-vein piggyback technique was the only approach to show a significant difference among revascularization districts, favoring the PDA territory. Anastomoses to the diagonal artery were limited to only two cases of artery-on-vein piggyback configurations, preventing a thorough analysis. There only flow distinctions observed concerning the other target coronary arteries occurred between the OM and PDA conduits of the vein-on-aorta configurations (*p* = 0.044).

## 4 Discussion

The results from our study combining clampless devices and the piggyback proximal anastomosis technique during OPCABG focused on postoperative outcomes and intraoperative TTFM data, with particular attention given to the potential advantages of reducing aortic manipulation.

Our analysis highlights three key benefits. First, adopting a single clampless aortic inflow for all the proximal anastomosis is safe and provides strong early mid-term outcomes. Second, by reducing aortic manipulation and minimizing additional holes, this approach significantly lowers the risk of stroke, offering an alternative strategy to anaortic OPCABG in patients with some degree of calcium in the ascending aorta, when anaortic technique is not achievable. Third, intraoperative TTFM demonstrated excellent flows, especially when arterial grafts were used with no postoperative myocardial infarctions.

These results on the piggyback technique are in line with previous reports: Yanagawa et al. described a single surgeon experience of 17 patients undergoing OPCABG with the piggyback technique aided with the Heartstring III Proximal Seal System (10). No graft failure or revisions were described, and TTFM showed no competitive flow between the two piggyback conduits. In a study by Hayashi et al., 175 piggyback anastomoses were carried out among 213 CABG patients where the RITA was employed (11). However, a specific analysis of the piggyback outcomes was not conducted. Finally, Hamasaki et al. described their cohort of 28 piggyback proximal anastomoses highlighting no cases of graft damage or bleeding at the anastomosis site, no strokes or other complications associated with proximal anastomosis (15).

### 4.1 Postoperative outcomes

Our findings revealed a very low incidence of postoperative strokes, affirming the hypothesis that reduced aortic manipulation minimizes cerebrovascular complications. Furthermore, we observed no postoperative myocardial infarctions, and the need for transfusion support was within acceptable limits. These outcomes suggest that our technique is both safe and effective in maintaining cardiac perfusion without increasing bleeding risks. With a low rate of readmissions and no in-hospital or 30-day mortality, the technique demonstrates favorable short-term survival and excellent postoperative results. Most importantly, the equal distribution of repeat revascularization among the piggyback grafts shows that these events are independent of the configuration.

### 4.2 Clinical implications and benefits

The combination of piggyback and clampless technique provides a valuable alternative for patients with high cerebrovascular risk or significant aortic disease. By reducing the number of aortic punctures, it decreases the risk of clinical stroke and embolization. Additionally, it offers mechanical advantages when using smaller arterial conduits, such as the radial artery or RITA, with a proximal inflow in the aorta. Direct proximal anastomoses of these conduits to the aorta carry risks, including graft kinking or flattening, which can compromise graft patency. By utilizing a single, well-supported anastomotic site, the piggyback method avoids these complications and enhances long-term graft functionality (10, 11, 15). These benefits underscore its potential as a safer, more streamlined approach, particularly for patients with complex aortic conditions.

Further supporting the efficacy of this technique, observations from redo-CABG procedures show that proximal anastomotic sites of old SVG often remain patent, even when the grafts themselves have occluded (16, 17). This finding highlights the durability of the proximal anastomotic site and reinforces the potential benefits of limiting aortic manipulation. By avoiding the so-called “Swiss cheese phenomenon”, where multiple punctures in the aorta are made for individual grafts, the piggyback technique offers a more streamlined and possibly safer approach with potential benefit in reducing the burden of SBI with clinical implications in the mid and long term outcomes of patients post CABG (6).

### 4.3 Limitations

This study has some limitations, including an early mid-term follow-up period, a relatively small cohort size, and a single-surgeon experience. Further multicenter studies with larger patient groups and extended follow-up are needed to confirm the long-term benefits of the clampless piggyback technique, particularly for graft patency and potential cognitive outcomes.

Standardized neuropsychological tests like objective measures of memory, attention and concentration, executive function, and processing speed and reaction time were not routinely incorporated to evaluate postoperative cognitive dysfunction (POCD). Therefore, the assessment may not have fully captured the subtle cognitive deficits associated with SBI and POCD.

### 4.4 Conclusion

The clampless piggyback proximal anastomosis technique demonstrates promising early mid-term outcomes in CABG patients, particularly in minimizing stroke risk and maintaining graft patency. The combination of venous and arterial conduits using this method appears to optimize flow, as evidenced by the TTFM data, while avoiding the pitfalls of traditional anastomosis techniques that involve multiple aortic punctures. The low incidence of major postoperative complications and favorable clinical outcomes support the continued use of this method, particularly in high-risk patients. Further studies with extended follow-up are warranted to confirm the long-term benefits of this technique.

## Data Availability

The data that supports the findings of this study are available upon reasonable request to the corresponding author, pending institutional approval. Requests to access the datasets should be directed to massimo.baudo@icloud.com.
